# The Association of Dietary Pattern with the Risk of Prehypertension and Hypertension in Jiangsu Province: A Longitudinal Study from 2007 to 2014

**DOI:** 10.3390/ijerph19137620

**Published:** 2022-06-22

**Authors:** Hui Xia, Yuhao Zhou, Yuanyuan Wang, Guiju Sun, Yue Dai

**Affiliations:** 1 Key Laboratory of Environmental Medicine and Engineering of Ministry of Education, Department of Nutrition and Food Hygiene, School of Public Health, Southeast University, Nanjing 210009, China; huixia@seu.edu.cn (H.X.); 220213987@seu.edu.cn (Y.Z.); wyypro@foxmail.com (Y.W.); 2 Institute of Food Safety and Assessment, Jiangsu Provincial Center for Disease Control and Prevention, Nanjing 210009, China

**Keywords:** dietary patterns, hypertension, prehypertension, factor analysis

## Abstract

Hypertension is the most common chronic disease and the primary risk factor for cardiovascular diseases. Prehypertension is closely related to a variety of cardiovascular disease risk factors during the development of hypertension. The objective of this study was to explore the relationship between dietary patterns and hypertension in Jiangsu Province. Specifically, we included the participants from 2007 and then followed up in 2014 in the Jiangsu Province of China and collected information from food frequency questionnaires, anthropometric measurements, and disease self-reports. A total of 1762 women and men were included in the final analysis. We extracted four dietary patterns using factor analysis, calculated the pattern-specific factor scores, and divided the scores into quartiles, which increased from Q1 to Q4. Compared with participants in Q1, an increased risk of high diastolic blood pressure was found in Q4 of the snack dietary pattern. Additionally, participants in Q2–Q4 of the frugal dietary pattern were found to have a positive association with abnormal blood pressure. However, the results found in the frugal dietary pattern vanished after adjusting more confounders in Q4 of high systolic blood pressure. We found that some food items were associated with hypertension and prehypertension. The overconsumption of salt and alcohol are risk factors for both prehypertension and hypertension. Added sugar and saturated fatty acids are risk factors for hypertension, which may provide suggestions for the residents in China to change dietary habits to prevent prehypertension and hypertension.

## 1. Introduction

Hypertension, one of the most common risk factors for cardiovascular disorders, profoundly affects the life quality of human beings and brings tremendous epidemiological and economic burdens to society and the families of patients. A systematic review and meta-analysis, including 46 analyses, showed that prolonged elevated blood pressure was positively linked to the risk of cardiovascular disease [1]. Recent research showed that between 1990 and 2019, global hypertension prevalence did not change substantially. However, despite the stability of global hypertension prevalence, the absolute number of hypertensive patients aged 30–79 years was doubled by the development of aging progress and growing population, from 331 million women and 317 million men in 1999 to 626 million women and 652 million men in 2019 [2]. According to the results of the China Hypertension Survey published in 2018, the crude prevalence of hypertension in people aged more than 18 was 27.9%, and the weighted prevalence was 23.2%. Based on the above data, 1 in every 4 adults had hypertension, and about 224 million people were suffering from hypertension [3]. Notably, prehypertension is of increasing interest to researchers. A cohort study proved that people with prehypertension were at twice the risk of developing high blood pressure compared to healthy people [4]. Prehypertension, defined as an intermediate area between normal blood pressure and hypertension, is defined as blood pressure >120/80 mmHg yet <140/90 mmHg, has become a common condition and can increase the incidence of hypertension and cardiovascular events. A representative sample that represents the total Chinese adult population showed that about 50.9% of adults aged 18 years and above without a history of hypertension had prehypertension [5]. A 15-year study in the 35–64-year-old population without baseline hypertension in China showed that among the prediction scores established by multiple risk factors for hypertension, SBP level of 130–139 mmHg had the highest risk score factor, which was significantly higher than obesity, family history, and age. DBP levels of 80–89 mmHg also had a significant impact on the risk of hypertension. More importantly, people with prehypertension had a significantly increased risk of cardiovascular disease [6]. A long-term cohort study in 2018 showed that 65% of people with blood pressure between 130/80 mmHg and 139/89 mmHg developed hypertension. After adjusting for all confounding factors, the risk of acute cardiovascular events in the last 20 years was 78% higher than that of the people with blood pressure <120/80 mmHg, and the mortality of cardiovascular disease was 150% higher than theirs [6]. Recently, the prevention strategy for hypertension in China has been mainly based on treatment, which failed to decrease the prevalence and even increased it across age groups [3]. Thus, a healthy lifestyle should be highly supported to adjust the prevention strategy to curb the incidence of hypertension and prehypertension [7,8,9].

Dietary patterns refer to presenting individual foods eaten in real life in the form of combinations and discussing the association between food combinations and disease and health. There are limitations in the traditional approaches, including failing to take into account the interaction and relationship between nutrients, detecting the small effects of single nutrients, and explaining how these nutrients act combinedly. Factor analysis, as a statistical method of dietary patterns, has been applied to identify patterns from collected dietary data and confirm relations between eating patterns and multiple health or disease outcomes, including metabolic syndrome, BMI, waist circumference, cancer, hypertension, diabetes, all-cause mortality, CVD, plasma, and other outcomes by reducing data into patterns based upon intercorrelations between dietary items [10,11,12,13,14,15,16]. This method can better reflect the actual situation, study the interaction between different nutrients or foods, and is more predictive for evaluating the real effect of diet on chronic diseases [17,18,19,20,21,22].

Jiangsu Province, which is located in the eastern coastal area of China, is an economically flourishing area with a population of 80.5 million. With the sustained and rapid development of the economy and technology, the lifestyle and diet of residents in Jiangsu Province have changed dramatically. Meanwhile, the prevalence of abnormal blood pressure has increased simultaneously in Jiangsu Province [23]. Moreover, the etiology of hypertension is still unclear. Therefore, this study aimed to investigate the associations between dietary patterns and hypertension and prehypertension in residents of Jiangsu Province by applying factor analysis.

## 2. Methods and Materials

### 2.1. Participants

This study was based on the National Nutrition and Health Survey of China, which has been carried out every 5 years since 2002 by a stratified multi-stage cluster sampling method. In the present study, we specifically included the participants in 2007 and then followed up in 2014 in the Jiangsu Province of China. Participants who met the following criteria were included, (1) 18.9 kg/m^2^ < BMI < 28 kg/m^2^; (2) with systolic blood pressure (SBP) < 130 mmHg and diastolic blood pressure (DBP) < 80 mmHg; (3) without other chronic diseases or family history diseases, such as hypertension, diabetes, hyperlipidemia and so on. A total of 1762 women and men aged from 18 to 86 years were included in the final analysis. The participants are all permanent residents of Jiangsu Province with poor mobility. Local eating patterns and habits are fixed. Meanwhile, the dietary habits of the participants were also fixed; thus, they could represent local eating habits. The Medical Ethics Committee of Jiangsu Provincial Center for Disease Control and Prevention approved this study, and consent forms were required from all participants.

### 2.2. Dietary Assessment and Other Covariates

A validated semi-quantitative food frequency questionnaire (FFQ) was sent in 2007 and 2014, respectively. The questionnaire included more than 100 food items categorized into staple foods, beans and their products, vegetables, mushrooms and algae, fruits, dairy products, meat and its products, eggs, seafood, snacks, cooking oil, condiments, and beverages. The response rate of the questionnaire was 90%. In addition, other covariates of sociodemographic and lifestyle were investigated as well, including age, gender, marital status, education level, smoking, drinking, weight, height, BMI, and waist circumference. 

### 2.3. Assessment of Hypertension

Blood pressure levels were measured 3 times after participants had rested for 5 min in a peaceful condition using a standard mercury sphygmomanometer [24]. The mean values were used for further analysis. We followed up and recorded the blood pressure levels of all participants in 2007 and 2014. Hypertension is defined by blood pressure and SBP ≥ 140 mmHg and/or DBP ≥ 90 mmHg. Prehypertension is defined as 120 mmHg ≤ SBP ≤139 mmHg or 80 mmHg ≤ DBP ≤ 89 mmHg.

### 2.4. Statistical Analysis

Categorical variables and continuous variables were presented as percentages and mean ± standard deviation, respectively. ANOVA was used in analyzing continuous variables, and the χ2 test was applied to compare the categorical data of participants. The factor analysis method was used to identify dietary patterns based on the response to the questionnaire. Four components were significant under the Kaiser criterion with Varimax orthogonal rotation (eigenvalue >1.2). Pattern-specific factor scores were calculated as the sum of the food factor loading coefficient and the standardized consumption of each food item associated with a specific pattern per week. Only the absolute value of the factor scores greater than 0.21 could be included in the analysis due to the strong relationship between these factors and the identified components. We calculated the pattern-specific factor scores for each dietary pattern and divided the scores into quartiles, which increased from Q1 to Q4. Multivariate logistic regression analysis was applied to calculate the odds ratio (OR) and the 95% confidence intervals (95% CIs) to estimate the association between dietary patterns and blood pressure abnormalities (high SBP, high DBP, prehypertension, and hypertension) after adjusting for covariates. All analyses were performed using SPSS 22.0 (IBM SPSS Inc., USA). All *p*-values were two-sided (α = 0.05). The dietary patterns were named according to the interpretability of the overall diet and the magnitude of the factor scores.

### 2.5. Ethics Approval and Consent to Participate

The study was approved by the Ethics Committee of the Jiangsu Provincial Center for Disease Control and Prevention, reference number JSCDC2014236. An informed consent form regarding this study was signed by all participants.

## 3. Results 

### 3.1. Basic Information of Population

A total of 1762 participants, including 817 (46.4%) males and 945 (53.6%) females aged 18–86 years, were included in this study. Among participants, the prevalence of systolic blood pressure was 60.6%. A total of 1067 participants, including 521 (63.8%) males and 546 (57.8%) females, had high SBP. The prevalence of high DBP was 56% (986/1762), including 501 (61.3%) males and 485 (51.3%) females. A total of 1237 (70.2%) participants, including 611 (78.4%) males and 626 (66.2%) females, were classified as having prehypertension or hypertension. 

### 3.2. Dietary Patterns and the Characteristics of the Individuals in Each Dietary Pattern

A meat-based dietary pattern, modern dietary pattern, snack dietary pattern, and frugal dietary pattern were identified separately by factor analysis. The suitability for factor analysis of the sample was diagnosed by the Kaiser–Meyer–Olkin index (0.675) and Bartlett’s test (*p* < 0.001). Four dietary patterns, which were named according to the food items showing high loading on each of the four patterns and the interpretability of the overall diet analyzed by factor analysis, are shown in Table 1. The demographic characteristics of all participants adjusted for educational level, marital status, income, BMI, central obesity, smoking status, and alcohol consumption according to quartiles of the dietary patterns scores extracted by factor analysis are presented in Table 2. 

The meat-based dietary pattern, which was associated with the consumption of meats, poultry, organ meat, seafood, vegetables, eggs, nuts, and alcoholic drinks, explained 9.79% of the variance. There were significant differences in age, height, weight, BMI, smoking and drinking status, and DBP between participants in Q4 and Q1–Q3 (*p* < 0.05). Subjects in Q4 tended to be younger, have a higher height and BMI, and be smokers and drinkers. The modern dietary pattern, which was associated with the consumption of desalted condiments, oils, soft beverages, salt, snacks, eggs, and vegetables, explained 9.029% of the variance. There was a significant difference in age between participants in Q4 and Q1–Q3 (*p* < 0.05). The snack dietary pattern, which was associated with the consumption of milk and milk products, tubers, starches and products, dried legumes and legume products, fruits, pastry snacks, eggs, and vegetables, explained 8.477% of the variance. There was a significant difference in high SBP between participants in Q4 and Q1–Q3. (*p* < 0.05). The frugal dietary pattern, which was associated with the consumption of cereals and cereal products, salted and preserved vegetables, salt, oils, eggs, alcohol, dried legumes, and legume products, explained 7.807% of the variance. There were significant differences in BMI, waistline, SBP, DBP, the proportion of overweight, obese, smokers, drinkers, high SBP, high DBP, hypertension, and prehypertension between participants in Q4 and Q1–Q3. 

### 3.3. Association between Dietary Patterns and Abnormal Blood Pressure

The OR of elevated blood pressure across quartiles of dietary patterns is indicated in Table 3. Participants in the fourth quartile of the snack dietary pattern tended to have higher DBP (compared to Q1, OR = 1.434, 95%CI: 1.068~1.925). After adjusting more confounders (age, sex, height, weight, smoking status, BMI, and calorigenic nutrients), the association still existed (OR = 1.442, 95%CI: 1.044~1.993). Moreover, higher quartiles of the frugal dietary pattern revealed an association with higher DBP, hypertension, and prehypertension in both model 1 and model 2. Meanwhile, compared with the lowest quartile, higher quartiles of the frugal dietary pattern had a positive relationship with higher SBP. However, this relationship between Q4 and Q1 vanished after adjusting more confounders (model 1: OR = 1.383, 95%CI: 1.059~1.808; model 2: OR = 1.063; 95%CI: 0.782~1.446). 

## 4. Discussion

In the present study, we extracted four kinds of dietary patterns in Jiangsu Province by the factor analysis method: meat-based dietary pattern, modern dietary pattern, snack dietary pattern, and frugal dietary pattern. According to the results of this study, specific dietary patterns, including snack dietary pattern and frugal dietary pattern, were associated with the risk of elevated blood pressure. Prehypertension is not only a transitional stage from normal blood pressure to hypertension but also a crucial stage in the prevention and treatment of hypertension.

Although it is currently recognized that prehypertension does not meet the diagnostic criteria of clinical hypertension, it has a harmful impact on the human body. Additionally, effective blood pressure control has been proven to be associated with the primary prevention and tertiary prevention of some cardiovascular diseases [25,26]. Therefore, healthy blood pressure is important for maintaining good health.

The meat-based pattern had no association with elevated blood pressure, which was inconsistent with previous studies. Several epidemiological studies suggest that intake of red meat adversely affects blood pressure [27,28,29]. A study of 41,541 American female nurses reported a positive association between red meat and elevated blood pressure [30]. Also, a trial of dietary approaches to stop hypertension (DASH) proved that reducing red meat in the diet can lower blood pressure [8]. However, a cohort study between 1991–2015 showed that a moderate intake of red meat was associated with a lower risk of elevated blood pressure [31]. Moreover, a clinical trial showed that low-sodium dietary approaches to stop the hypertension-type diet, including lean red meat, can lower blood pressure [32]. The fat content of red meat might play an important role in affecting blood pressure. As we all know, animal fat, which has massive saturated fatty acid, is positively correlated with elevated blood pressure. The effect of animal meat in this study remained unclear. Meanwhile, high consumption of alcoholic drinks also showed a positive association with blood pressure. A systematic review and meta-analysis found that reducing alcohol intake in people who drink more than two drinks each day would reduce the disease burden brought by alcohol consumption and high blood pressure [33]. A plausible explanation was that the high intake of vegetables, nuts, and seafood, which contained unsaturated fatty acids and micronutrients, might contribute to reducing the risk of abnormal blood pressure [34,35]. The modern dietary pattern was not correlated with the risk of elevated blood pressure. This is counterintuitive since a high intake of condiments, including soy sauce and salt, is correlated with elevated blood pressure. Dietary plant oil, milk, and its products may offset the adverse cardiovascular effects of soy sauce and salt, which might be another explanation for this pattern and hypertension.

We found the snack dietary pattern is positively correlated with high elevated DBP (in Q4) both in model 1 and model 2. The phenomenon that a high intake of vegetables, fruit, and dairy products was positively associated with abnormal blood pressure was different from the result of the previous study [36]. A systematic review and meta-analysis that included 9 cohorts demonstrated that there was an inverse dose-response relationship between the risk of hypertension and fruit and vegetable intake [37]. Also, a prospective cohort study of the general Chinese population showed a significant inverse association between egg consumption and CVD [38]. Moreover, legumes and their products were found to have an inverse association with hypertension [39]. The positive association between this pattern and high DBP could be attributed to the high consumption of pastry snacks. Most pastry snacks, which were made up of plants, animal products, or dairy products, belonged to ultra-processed foods. Ultra-processed foods have a high content of sodium, trans-fatty acids, added sugar, refined starches, and calories and are low in nutrients and dietary fiber. Excessive intake of pastries and snacks would put the body in an unhealthy state. According to the conclusion of previous studies, a high intake of pastry snacks could significantly increase the risk of abnormal blood pressure [40,41]. Several countries have advised consumers to limit the consumption of ultra-processed foods which are full of added sugar and saturated fat to decrease the risk of cardiovascular disease. 

The frugal dietary pattern was found to have a positive association with hypertension and prehypertension. This was consistent with the previous study in Jiangsu Province [42]. Salted and preserved vegetables contain a lot of salt, and excessive intake of this food would lead to excessive salt intake, which was positively associated with the prevalence of hypertension. Excessive intake of salt has become the main factor for the development of hypertension. The increase in salt intake would lead to an increased extracellular fluid volume which would result in elevated blood pressure. A meta-analysis including 17 RCTs showed that reducing salt intake could lower the blood pressure of both healthy and hypertensive individuals [43]. The World Health Organization recommends that the daily salt intake should be less than 5 g [44]. However, the Chinese people’s diet is characterized by high sodium and low potassium. The average daily salt intake of northern Chinese residents is 12–15 g, and that of southern residents is 8–10 g, which is far beyond the WHO’s recommendation. Previous studies have proved that more than 50% of hypertension patients are sodium-sensitive [45]. Meanwhile, dietary research in Chinese adults demonstrates that people with high salt sensitivity have a higher risk of increased blood pressure. Sodium reduction in the daily diet can lower blood pressure and the risk of cardiovascular disease [46,47,48,49]. Additionally, excessive consumption of alcohol is a risk factor for hypertension. Several studies have demonstrated that alcohol, even a light intake, is associated with an increased risk of hypertension [50,51,52,53]. If the daily intake of alcohol is more than 36 g, the systolic and diastolic blood pressure will increase by 3.5 mmHg and 2.1 mmHg on average, respectively, and alcohol consumption has a positive association with the increase.

To our knowledge, this was the first study to identify the dietary patterns of residents in Jiangsu Province and explore their relationships with the incidence of prehypertension and hypertension. Additionally, this study was designed as a prospective observational study, which was needed to ascertain the causality. Meanwhile, four dietary patterns in this study were determined by factor analysis, which was a better reflection of real eating habits than patterns based on previously adopted dietary assumptions. However, these dietary patterns were sample-specific and might be inapplicable to the population in other areas. In addition, although we tried to control the confounding factors, they might not be totally removed due to the complicated relationships in food. Moreover, we did not collect individual information on physical activity, which may affect the incidence of high blood pressure. Finally, we did not measure blood pressure yearly, which has made it unclear how many years of that specific dietary pattern has caused hypertension or prehypertension. Future research should therefore concentrate on the investigation of annual blood pressure data.

In conclusion, this study showed evidence that excessive intake of salt, alcohol, added sugar, and saturated fatty acids were significantly associated with a high prevalence of prehypertension and hypertension among residents in Jiangsu Province. People need to change their habits to prevent increased blood pressure.

## 5. Conclusions

Different dietary patterns do affect blood pressure in adults in Jiangsu Province. The diets of adults in the Jiangsu region have undergone dramatic changes in recent years. We identified dietary patterns in this region and found that frugal dietary pattern and snack dietary pattern were associated with a higher prevalence of prehypertension and hypertension among adults in Jiangsu Province, China.

## Figures and Tables

**Table 1:** The factors of dietary patterns identified by factor analysis in the participants.

Food Groups	Meat-Based Pattern	Modern Pattern	Snack Pattern	Frugal Pattern
Cereals and cereal products	−0.110	0.042	−0.118	0.563 ^a^
Tubers, starches and products	0.032	−0.111	0.512 ^a^	0.128
Meat	0.697 ^a^	−0.018	0.042	−0.111
Poultry	0.686 ^a^	0.010	0.062	−0.096
Organ meat	0.463 ^a^	−0.025	−0.020	−0.003
Seafood	0.569 ^a^	0.142	0.144	0.035
Milk and milk products	−0.067	0.211 ^a^	0.532 ^a^	−0.277 ^a^
Eggs and egg products	0.212 ^a^	0.041	0.420 ^a^	0.327 ^a^
Dried legumes and legume products	0.063	−0.137	0.522 ^a^	0.267 ^a^
Vegetables	0.307 ^a^	0.082	0.352 ^a^	0.006
Salted and preserved vegetables	−0.063	−0.068	0.076	0.511 ^a^
Fruit	0.189	0.056	0.494 ^a^	−0.214 ^a^
Nuts	0.312 ^a^	0.133	0.129	−0.042
Snacks	−0.014	0.381 ^a^	0.161	−0.315 ^a^
Pastry snacks	0.018	0.134	0.481 ^a^	−0.219 ^a^
Alcoholic beverage	0.287 ^a^	0.030	0.024	0.278 ^a^
Fats and oils	−0.037	0.565 ^a^	0.036	0.398 ^a^
Salt	−0.118	0.478 ^a^	−0.086	0.466 ^a^
Desalted condiments	0.202	0.791 ^a^	0.063	0.059
Soft beverages	0.166	0.579 ^a^	−0.029	−0.195

^a^: the absolute value of factor score ≥0.21.

**Table 2:** Baseline characteristics of participants by quartiles of dietary patterns in the participants.

Patterns	Meat-Based Pattern				Modern Pattern				Snack Pattern				Frugal Pattern			
	Q1(509)	Q2(482)	Q3(374)	Q4(397)	Q1(387)	Q2(615)	Q3(418)	Q4(342)	Q1(395)	Q2(675)	Q3(343)	Q4(349)	Q1(439)	Q2(440)	Q3(444)	Q4(439)
Age	51.7 ± 13.9	51.1 ± 12.6	49.5 ± 12.5	**47.0 ± 12.9 ^a^**	53.0 ± 12.6	50.1 ± 12.8	49.5 ± 13.2	**47.0 ± 13.7 ^a^**	50.4 ± 12.8	50.2 ± 12.6	49.6 ± 13.5	49.4 ± 14.3	47.2 ± 15.0	51.0 ± 12.7	50.5 ± 12.9	**51.3 ± 11.5 ^a^**
sex(%)																
Male	183(22.4)	207(25.3)	184(22.5)	243(29.7)	204(25.0)	275(33.7)	184(22.5)	154(18.8)	185(22.6)	313(38.3)	145(17.7)	174(21.3)	150(18.4)	177(21.7)	248(30.4)	242(29.6)
Female	326(34.5)	275(29.1)	190(20.1)	154(16.3)	183(19.4)	340(36.0)	234(24.8)	188(19.9)	210(22.2)	362(38.3)	198(21.0)	175(18.5)	289(30.6)	263(27.8)	196(20.7)	197(20.8)
height(cm)	160.0 ± 8.3	161.0 ± 7.8	161.4 ± 8.5	**163.8 ± 7.5 ^a^**	161.3 ± 7.8	161.2 ± 8.7	161.4 ± 8	161.9 ± 7.7	161.5 ± 8.5	161.3 ± 8.2	161.2 ± 7.8	161.8 ± 8.1	160.2 ± 8.1	160.1 ± 8.0	162.5 ± 8.4	**162.9 ± 7.7 ^a^**
weight(kg)	61.4 ± 10.3	61.4 ± 10.2	61.0 ± 10.3	**63.3 ± 10.5**	61.8 ± 10.9	61.7 ± 10.2	61.3 ± 10.2	62.4 ± 10.2	62.5 ± 10.2	61.5 ± 10.3	61.7 ± 10.6	61.4 ± 10.4	59.9 ± 9.9	61.0 ± 10.1	62.3 ± 11.2	**63.8 ± 9.7 ^a^**
Waist circumstance(cm)	81.5 ± 10.3	80.9 ± 10.2	80.3 ± 10.3	81.0 ± 10.5	81.1 ± 9.8	81.1 ± 9.6	80.3 ± 9.7	81.5 ± 9.2	81.6 ± 9.1	80.7 ± 9.4	80.6 ± 10.4	81.0 ± 9.6	78.8 ± 9.4	80.9 ± 10	81.4 ± 9.7	**82.7 ± 8.9 ^a^**
BMI(kg/m^2^)	24.1 ± 3.8	23.7 ± 3.3	23.4 ± 3.1	**23.5 ± 3.2**	23.7 ± 3.6	23.7 ± 3.4	23.5 ± 3.3	23.8 ± 3.3	24.0 ± 3.5	23.6 ± 3.3	23.7 ± 3.5	23.4 ± 3.3	23.3 ± 3.1	23.8 ± 3.6	23.5 ± 3.3	**24.1 ± 3.5 ^a^**
BMI grade(%)																
Light	15(24.6)	17(27.9)	17(27.9)	12(19.7)	11(18.0)	15(24.6)	23(37.7)	12(19.7)	11(18.0)	24(39.3)	8(13.1)	18(29.5)	16(26.2)	19(31.1)	18(29.5)	8(13.1)
Normal	258(27.6)	250(26.8)	202(21.6)	224(24.0)	211(22.6)	334(35.8)	220(23.6)	169(18.1)	207(22.2)	348(37.3)	182(19.5)	197(21.1)	247(26.4)	220(23.6)	251(26.9)	216(23.1)
Overweight	173(29.2)	165(27.9)	126(21.3)	128(21.6)	128(21.6)	208(35.1)	133(22.5)	123(20.8)	130(22.0)	249(42.1)	114(19.3)	99(16.7)	140(23.6)	157(26.5)	128(21.6)	**167(28.2) ^a^**
Obesity	63(36.0)	50(28.6)	29(16.6)	33(18.9)	37(21.1)	58(33.1)	42(24.0)	38(21.7)	47(26.9)	54(30.9)	39(22.3)	35(20.0)	36(20.6)	44(25.1)	47(26.9)	**48(27.4) ^a^**
Central obesity(%)																
Yes	158(34.6)	132(28.9)	83(18.2)	**84(18.4) ^a^**	102(22.3)	155(33.9)	105(23.0)	95(20.8)	105(23.0)	169(37.0)	96(21.0)	87(19.0)	96(21.0)	118(25.8)	114(24.9)	129(28.2)
No	351(26.9)	350(26.8)	291(22.3)	313(24.0)	285(21.8)	460(35.2)	313(24.0)	247(18.9)	290(22.2)	506(38.8)	247(18.9)	262(20.1)	343(26.3)	322(24.7)	330(25.3)	310(23.8)
Smoke(%)																
Yes	94(18.3)	121(23.6)	123(24.0)	**175(34.1) ^a^**	131(25.5)	168(32.7)	121(23.6)	93(18.1)	113(22.0)	204(39.8)	88(17.2)	108(21.1)	82(16.0)	118(23.0)	166(32.4)	**147(28.7) ^a^**
No	415(33.2)	361(28.9)	251(20.1)	222(17.8)	256(20.5)	447(35.8)	297(23.8)	249(19.9)	282(22.6)	471(37.7)	255(20.4)	241(19.3)	357(28.6)	322(25.8)	278(22.3)	292(23.4)
Drink (%)																
Yes	52(15.4)	87(25.7)	74(21.9)	**125(37.0) ^a^**	93(27.5)	108(32.0)	76(22.5)	61(18.0)	81(24.0)	121(35.8)	66(19.5)	70(20.7)	37(10.9)	69(20.4)	101(29.9)	**131(38.8) ^a^**
No	457(32.1)	395(27.7)	300(21.1)	272(19.1)	294(20.6)	507(35.6)	342(24.0)	281(19.7)	314(22.1)	554(38.9)	277(19.5)	279(19.6)	402(28.2)	371(26.1)	343(24.1)	308(21.6)
SBP(mmHg)	125.4 ± 18.9	127 ± 19.3	125.4 ± 19.5	125.4 ± 20.4	127.5 ± 20.0	126.2 ± 19.1	124.6 ± 19.4	124.9 ± 19.6	125.8 ± 20.0	125.7 ± 18.9	125.6 ± 19.1	126.4 ± 20.6	122.1 ± 19.0	128.2 ± 20.3	127.5 ± 19.4	**125.5 ± 18.9 ^a^**
DBP(mmHg)	79.4 ± 10.9	80.6 ± 10.2	81.1 ± 10.3	**81.2 ± 11.4 ^a^**	80.7 ± 10.6	80.6 ± 10.9	80.2 ± 10.2	80.6 ± 11.2	80.1 ± 11.2	80.5 ± 10.6	79.7 ± 10.3	81.8 ± 10.8	78.2 ± 11.1	81.7 ± 10.2	81.4 ± 10.7	**80.8 ± 10.6 ^a^**
High SBP(%)																
Yes	309(29.0)	302(28.3)	223(20.9)	233(21.8)	239(22.4)	389(36.5)	239(22.4)	200(18.7)	235(22.0)	416(39.0)	207(19.4)	209(19.6)	229(21.5)	287(26.9)	287(26.9)	**264(24.7) ^a^**
No	200(28.8)	180(25.9)	151(21.7)	164(23.6)	148(18.0)	226(24.6)	179(37.7)	142(19.7)	160(23.0)	259(37.3)	136(19.6)	140(20.1)	210(30.2)	153(22.0)	157(22.6)	175(25.2)
High DBP(%)																
Yes	263(26.7)	277(28.1)	218(22.1)	228(23.1)	218(22.1)	349(35.4)	220(22.3)	199(20.2)	217(22.0)	376(38.1)	171(17.3)	**222(22.5)**	205(20.8)	269(27.3)	258(26.2)	**254(25.8) ^a^**
No	246(31.7)	205(26.4)	156(20.1)	169(21.8)	169(21.8)	266(34.3)	198(25.5)	143(18.4)	178(22.9)	299(38.5)	172(22.2)	127(16.4)	234(30.2)	171(22.0)	186(24.0)	185(23.8)
Hypertension and prehypertension(%)																
Yes	343(27.7)	347(28.1)	265(21.4)	282(22.8)	273(22.1)	441(35.7)	284(23.0)	239(19.3)	274(22.2)	476(38.5)	230(18.6)	257(20.8)	270(21.8)	326(26.4)	326(26.4)	**315(25.5) ^a^**
No	166(31.6)	135(25.7)	109(20.8)	115(21.9)	114(22.1)	174(35.4)	134(22.3)	103(20.2)	121(23.0)	199(37.9)	113(21.5)	92(17.5)	169(32.2)	114(21.7)	118(22.5)	124(23.6)

Adjusted education level, marital status, income status, BMI, central obesity, smoking, drinking. ^a^: compared with Q1~Q3, *p*_Q4_ < 0.05.

**Table 3:** The OR of elevated blood pressure across quartiles of dietary patterns.

Outcome	Dietary Pattern	Model	Q1	Q2	Q3	Q4
				OR(95%CI)	OR(95%CI)	OR(95%CI)
High SBP	Meat-based dietary pattern	model 1	1.000	1.086(0.840~1.403)	0.956(0.728~1.255)	0.920(0.704~1.202)
		model 2	1.000	1.141(0.859~1.514)	1.051(0.768~1.439)	1.045(0.760~1.436)
	Modern pattern	model 1	1.000	1.066(0.820~1.386)	0.827(0.624~1.096)	0.872(0.648~1.174)
		model 2	1.000	1.254(0.946~1.663)	1.013(0.745~1.377)	1.152(0.830~1.599)
	Snack pattern	model 1	1.000	1.094(0.848~1.410)	1.036(0.771~1.392)	1.016(0.758~1.363)
		model 2	1.000	1.121(0.853~1.474)	1.044(0.756~1.442)	1.219(0.878~1.694)
	Frugal pattern	model 1	1.000	1.720(1.312~2.256) ^a^	1.676(1.280~2.196) ^a^	1.383(1.059~1.808) ^a^
		model 2	1.000	1.477(1.101~1.981) ^a^	1.514(1.127~2.035) ^a^	1.063(0.782~1.446)
High DBP	Meat-based dietary pattern	model 1	1.000	1.264(0.984~1.624)	1.307(0.999~1.711)	1.262(0.969~1.643)
		model 2	1.000	1.284(0.976~1.690)	1.331(0.981~1.806)	1.149(0.842~1.567)
	Modern pattern	model 1	1.000	1.017(0.787~1.315)	0.861(0.652~1.137)	1.079(0.804~1.448)
		model 2	1.000	1.137(0.864~1.495)	0.939(0.695~1.267)	1.144(0.830~1.576)
	Snack pattern	model 1	1.000	1.032(0.804~1.324)	0.816(0.610~1.090)	1.434(1.068~1.925) ^a^
		model 2	1.000	0.982(0.752~1.282)	0.725(0.530~0.994)	1.442(1.044~1.993) ^a^
	Frugal pattern	model 1	1.000	1.796(1.373~2.348) ^a^	1.583(1.214~2.066) ^a^	1.567(1.201~2.046) ^a^
		model 2	1.000	1.814(1.363~2.416) ^a^	1.581(1.185~2.109) ^a^	1.395(1.031~1.888) ^a^
Hypertension and prehypertension	Meat-based dietary pattern	model 1	1.000	1.244(0.948~1.632)	1.177(0.880~1.572)	1.187(0.892~1.579)
		model 2	1.000	1.335(0.989~1.801)	1.328(0.951~1.855)	1.306(0.931~1.833)
	Modern pattern	model 1	1.000	1.058(0.800~1.401)	0.885(0.656~1.195)	0.969(0.705~1.332)
		model 2	1.000	1.281(0.947~1.733)	1.082(0.780~1.502)	1.203(0.847~1.709)
	Snack pattern	model 1	1.000	1.056(0.806~1.384)	0.899(0.659~1.226)	1.234(0.896~1.699)
		model 2	1.000	1.044(0.780~1.396)	0.836(0.595~1.174)	1.379(0.965~1.969)
	Frugal pattern	model 1	1.000	1.790(1.343~2.385) ^a^	1.729(1.300~2.300) ^a^	1.590(1.198~2.110) ^a^
		model 2	1.000	1.627(1.195~2.217) ^a^	1.620(1.186~2.212) ^a^	1.285(0.928~1.781) ^a^

^a^: *p* < 0.05 in logistic regression analysis. Model 1 has adjusted education level, marital status, income status, BMI, central obesity, smoking, and drinking. Model 2 has adjusted age, gender, marital status, income status, education level, smoking, drinking, weight, height, BMI, and waist circumference.

## Data Availability

Not applicable.
